# Ligands of Therapeutic Utility for the Liver X Receptors

**DOI:** 10.3390/molecules22010088

**Published:** 2017-01-05

**Authors:** Rajesh Komati, Dominick Spadoni, Shilong Zheng, Jayalakshmi Sridhar, Kevin E. Riley, Guangdi Wang

**Affiliations:** Department of Chemistry and RCMI Cancer Research Center, Xavier University of Louisiana, New Orleans, LA 70125, USA; rkomati@xula.edu (R.K.); dspadoni@xula.edu (D.S.); szheng@xula.edu (S.Z.); jsridhar@xula.edu (J.S.); kriley3@xula.edu (K.E.R.)

**Keywords:** liver X receptors, LXRα, LXRβ specific ligands, atherosclerosis, diabetes, Alzheimer’s disease, cancer, lipid metabolism, molecular modeling, interaction energy

## Abstract

Liver X receptors (LXRs) have been increasingly recognized as a potential therapeutic target to treat pathological conditions ranging from vascular and metabolic diseases, neurological degeneration, to cancers that are driven by lipid metabolism. Amidst intensifying efforts to discover ligands that act through LXRs to achieve the sought-after pharmacological outcomes, several lead compounds are already being tested in clinical trials for a variety of disease interventions. While more potent and selective LXR ligands continue to emerge from screening of small molecule libraries, rational design, and empirical medicinal chemistry approaches, challenges remain in minimizing undesirable effects of LXR activation on lipid metabolism. This review provides a summary of known endogenous, naturally occurring, and synthetic ligands. The review also offers considerations from a molecular modeling perspective with which to design more specific LXRβ ligands based on the interaction energies of ligands and the important amino acid residues in the LXRβ ligand binding domain.

## 1. Structure and Functions of Liver X Receptors

Nuclear receptors (NRs) are one of the most abundant classes of transcriptional regulators in animals. They regulate diverse biological functions including homeostasis, reproduction, development and metabolism, in normal as well as in pathological settings. Nuclear receptors are also known as hormone receptors (HRs) that are ligand-activated transcription factors, providing a direct link between signaling molecules that control these processes and transcriptional responses [1]. In all, NRs comprise a group of 48 ligand-activated transcription factors in humans.

The liver X receptors (LXRs) are NRs that act as oxysterol sensors, regulating genes involved in cholesterol and lipid metabolism. Based on the coding genes LXRs are classified as LXRα (NR1H3) and LXRβ (NR1H2) [2]. LXRα is expressed most highly in the liver and to a lesser extent in the kidney, small intestine, spleen, and adrenal gland [3,4]. In contrast, LXRβ is ubiquitously expressed [5].

LXRα consists of 447 [4] and LXRβ contains 461 [6] amino acids. The LXR molecules can be viewed as having four functional domains: (1) an amino-terminal ligand-independent activation function domain (AF-1), which may stimulate transcription in the absence of ligand; (2) a DNA-binding domain (DBD) containing two zinc fingers; (3) a hydrophobic ligand-binding domain (LBD) required for ligand binding and receptor dimerization, and; (4) a carboxy-terminal ligand-dependent transactivation sequence (also referred to as an activation function-2 (AF-2) domain) that stimulates transcription in response to ligand binding [7]. The DBD and LBD regions of LXRα and LXRβ have 75.6% and 74% sequence identity, respectively [8]. The LBD of LXRα features a three-layered α-helical sandwich structure common to all known nuclear receptors and contains ten α-helices [9]. Both LXRα and LXRβ function as heterodimers with the retinoid X receptor (RXR). LXR/RXR is a “permissive heterodimer” that may be activated by either LXR agonist or 9-*cis* retinoic acid, a specific RXR ligand. The LXR/RXR complex binds to an LXR responsive element (LXRE) in the promoter region of target genes which consists of two direct repeats of hexameric nucleotides, AGGTCA, separated by four or one nucleotide(s) (DR4 or DR1) [10]. It is through these target genes that LXR regulate various biological processes that are implicated in normal as well as pathological functions. Numerous LXR target genes have been identified such as ATP binding cassette (ABC) transporter isoforms A1, G1, G5, and G8, apolipoprotein E (ApoE), cholesteryl ester transfer protein (CETP), fatty acid synthase (FAS), cytochrome P450 isoform 7A1 (CYP7A1)—cholesterol 7α-hydroxylase, and carbohydrate regulatory element binding protein (ChREBP) [11].

**Figure 1:**
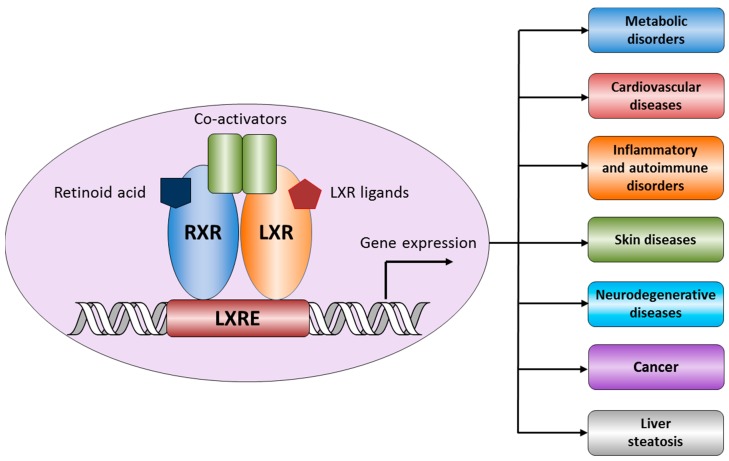
Liver X Receptors may be related to various pathological conditions.

The main function of LXRs is the regulation of cholesterol metabolism. Agonists of LXRs increase insulin sensitivity and stimulate insulin secretion. Activation of LXRs inhibits inflammation and autoimmune reactions. Moreover, pharmacological studies and genetic manipulations indicate that LXRs inhibit atherogenesis [12]. LXRs are also involved in the regulation of renin secretion [13], inhibition of amyloid b formation in the central nervous system [14], regulation of gonadal function and steroidogenesis both in gonads and in adrenals [15], proliferation and differentiation of keratinocytes [16], and inhibition of tumor cells proliferation [17]. LXR regulation of transcription activity can be explained by four different models- ligand independent repression, direct activation, ligand dependent activation and trans-repression. The specific activation/repression of gene transcription by LXRs is cell and gene dependent. In the absence of an agonist, the LXR-RXR heterodimer inhibits transcription by the recruitment of co-repressors N-CoR (nuclear receptor corepressor) and SMRT (silent mediator of retinoic acid receptor and thyroid receptor) [10,18]. Ligand binding initiates the dissociation of the co-repressors resulting in a moderate activation leading to stimulation of transcription. Ligand binding is followed by recruitment of co-activators and in this state the transcription levels are the highest. In the trans-repression model, LXRs have the capability of negatively regulating the expression of inflammatory genes. While the mechanism of such trans-repression is not completely understood, the process is known to involve the inhibition of inflammatory responses to cytokines via blockade of the activity of the signal transducer and transcription activator nuclear factor NF-κB, and activator protein 1 that induce transcription of the proinflammatory genes COX2, MMP9, IL-6, MCP-1, iNOS, IL-1β [19,20,21,22,23,24]. Consequently, alterations in endogenous LXR activity is evidenced in many pathological conditions such as atherosclerosis, cancer, neurological disorders such as multiple sclerosis, Alzheimer disease and Parkinson disease, arthritis and skin diseases (Figure 1).

### 1.1. LXR and Atherosclerosis

The physiological ligands of LXR are the oxysterols that are produced endogenously by enzymatic reactions, by reactive oxygen species (ROS)-dependent oxidation of cholesterol and by the alimentary processes. The high affinity of oxysterols to LXRs has defined their physiological role as “cholesterol sensors”. The ligand bound LXRs increase the expression of target genes associated with reverse cholesterol transport, cholesterol conversion to bile acid, and intestinal cholesterol absorption. Some of these genes are the ATP-binding cassette (ABC) transporters A1 and G1, the sterol response element-binding protein-1c (SREBP-1c), the apolipoprotein E, phospholipid transport protein, cholesterol 7α-hydroxylase and several other genes involved in lipogenesis such as FAS and stearoyl-CoA desaturase (SCD) [25,26,27,28,29,30,31]. LXRα-knockout mice on high cholesterol diet when subjected to genetic studies showed defects in cholesterol metabolism in the liver and a corresponding increase in plasma cholesterol levels indicating the therapeutic role of LXR agonists in atherosclerosis. Treatment with LXR agonists resulted in attenuation of atherosclerosis in vivo with a reduction in total cholesterol and/or elevation in high density cholesterol [28,32,33,34,35,36,37]. The upregulation of the lipogenesis genes by LXR increased plasma and hepatic triglyceride (TG) levels in mice and lipid accumulation in human mature adipocytes. Studies using LXR agonists have shown that the antiatherosclerotic effects are accompanied with either an increase in TG levels or with no effects on lipogenesis leading to the suggestion that some of the LXR agonists may be exhibiting antiatherosclerotic effect independent of lipogenesis through direct actions on vascular wall. Indeed, LXR agonists are negative regulators of key inflammatory genes TNFa, IL-1b, IL-6, COX2, iNOS and NF-κB leading to modulation of the atherosclerotic plaque/lesions and a reversal of plaque accumulation [19,20,21,22,38,39].

### 1.2. LXR and Cancer

LXR ligands were initially shown to have antiproliferative effects on prostate cancer cells. Similar effects were also evidenced in breast cancer cells, colorectal cancer cells and chronic lymphocytic leukemia. A more subtle effect was seen on acute myeloid leukemia cells. The antiproliferative effect of LXR ligands can be attributed to its role in lipid metabolism and inflammation/immunity. Cholesterol has been shown to accumulate in prostate tumor cells in increasing levels with a concomitant increase in the enzymes of the mevalonate pathway. This is attributed to the upregulation of the HGM-CoA reductase leading to de novo cholesterol synthesis which is an essential component for tumor growth. LXR activation mitigated cholesterol homeostasis leading to inhibition of proliferation in LNCaP prostate cancer cells [40,41]. Treatment of cancer cells with LXR ligands increased p27 protein (a cyclin dependent kinase inhibitor) levels and decreased S-phase kinase associated protein (SKP2), leading to an arrest in the cell cycle [40,42]. Thus exposure of ovarian cancer cells to the LXR ligands induced apoptosis. In breast cancer cells, the levels of p27 and p21 did not change upon LXR ligand treatment, but the SKP2 transcript and protein levels were decreased [22,43]. Evidence was found that LXR ligand treatment decreased the most important proliferative factor ERα transcript and protein levels in ER+ breast cancer cells [44].

While LXR agonists induce the expression of VEGF in murine/human primary macrophages and in murine adipose tissue, the basal expression of VEGF does not seem to involve LXRs as evidenced by the absence of vascular problems in LXR−/− mice. However, the tumor microenvironment is affected by the LXR ligand treatment wherein the endothelial cells showed disturbances in vascular endothelial growth factor receptor 2 distribution/signaling as related to angiogenesis with concomitant reduction in tubulogenesis, proliferation and cell migration [45,46]. This anti-angiogenic effect could be a result of LXR effect on endothilial cholesterol homeostasis that mediates an impaired VEGFR2 compartmentation and signaling. Thus changes in endothelial cholesterol have an effect on VEGFR2 signaling due to the interactions of LXR with the complex signaling pathways within the lipid rafts/caveolae. LXRβ is known to upregulate the expression of ApoE that is associated with inhibition of angiogenesis and metastatic invasion in cancer cells. Indeed, LXR ligand treatment of MCF7 breast cancer cells and melanoma cells showed an increase in ApoE leading to tumor growth suppression [47,48].

### 1.3. LXR and Alzheimer’s Disease (AD)

ApoE is the main lipid transporter protein in the central nervous system (CNS) [49]. It has been proposed that the ApoE protein associates with lipid particles and transports them both within and out of the CNS by acting as carriers for amyloid peptide transport. LXRs are known to regulate the expression of ApoE and ABCA1 in astrocytes thereby controlling the overall expression of ApoE and its lipidation levels. Recently, it has been shown that treatment with LXR agonists leads to upregulation of ApoE and ABCA1 resulting in the clearance of amyloid by promoting Aβ transport in Alzheimer’s disease [14,50,51,52]. The development of an LXR ligand that can penetrate the brain will have enormous therapeutic potential by itself or in combination with other AD therapeutics [53,54].

## 2. Ligands of Liver X Receptors

### 2.1. Endogenous Agonists

**Figure 2:**
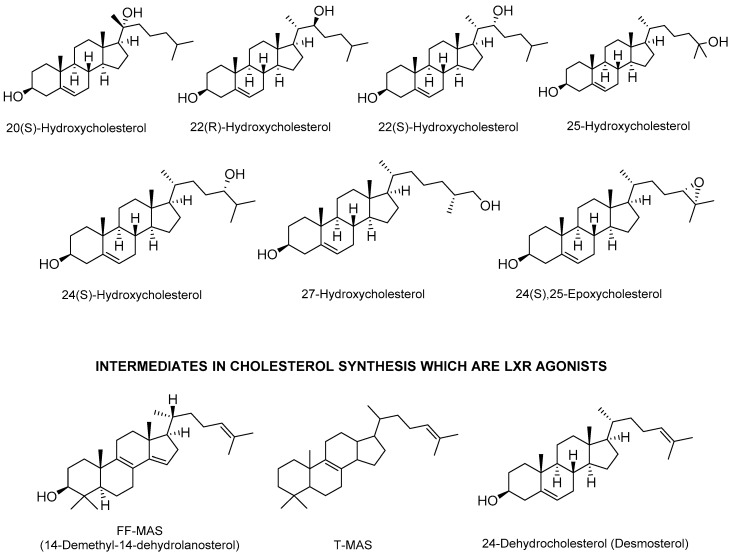
Representative endogenous ligands that activate LXRs.

Oxysterols (Figure 2) were found to be endogenous ligands for LXRs in mammals comprising two different ligand types that activate LXRs [55,56]. The first type which comes under oxidized derivatives of oxysterol includes 20(*S*)-, 22(*R*)-, 24(*S*)-, 25- and 27-hydroxy cholesterol and 24(*S*), 25-epoxycholesterols. These oxysterols bind to LXRs with *K*_d_ values ranging from 0.1 to 0.4 µM. 

24(*S*)-hydroxycholesterol, also known as cerebrosterol because of its abundance in brain, is synthesized by 24-hydroxylase. It was proven to be an efficient activator of LXR regulated gene ABCA1 [57,58]. 27-hydroxycholesterol is generated from cholesterol by the P450 enzyme sterol 27-hydroxylase which is encoded by Cyp27a1. It is further oxidized to aldehyde and carboxylic acid (cholestenoic acid) by 27-hydroxylase. Both the 27-hydroxycholesterol and cholestenoic acid are the ligands for LXRs [59,60,61]. 25-hydroxycholesterol, synthesized by 25-hydroxylase, is a potent regulator of LXR mediated pathways. It also influences the expression of LXR dependent genes LPL, ABCG5 and ABCG8 [62].

The second types of LXR activating oxysterols are the intermediates of cholesterol biosynthesis. 24(*S*), 25-epoxycholesterol is a unique oxysterol which is produced in a shunt of the mevalonate pathway. The loss of 24(*S*),25-epoxycholesterol decreases the expression of LXR target genes ABCA1 [63]. Other intermediates such as desmosterol and zymosterol also activates the LXR [64].

Meiosis activating sterols have been reported to stimulate the oocyte meiosis via LXR activation [37]. Examples include the sterol 4,4-dimethyl-5ax-cholesta-8,14,24-trien-31i-ol (FF-MAS), which is the intermediate of cholesterol synthesis generated in the ovaries and a closely related C29-sterol (4,4-dimethyl-5a-cholesta-8,24-dien-3p-ol) (T-MAS) [65].

### 2.2. Endogenous Antagonists

**Figure 3:**
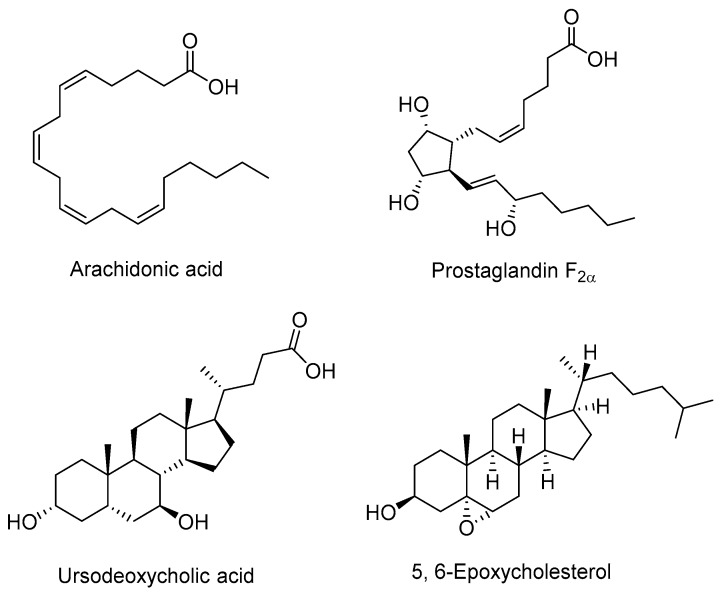
Representative endogenous ligands that inhibit LXRs (antagonists).

Several endogenous LXR antagonists have also been identified (Figure 3). Arachidonic acid and other fatty acids competitively inhibited the activation process of endogenous SREBP-1c gene by an external ligand T0901317 in cultured rat hepatoma cells. Arachidonate was shown to block the activation of a synthetic LXR-dependent promoter in transfected human embryonic kidney 293-cells. In vitro*,* arachidonate and other unsaturated fatty acids competitively blocked activation of LXR, which is reflected in a fluorescence polarization assay that measured ligand-dependent binding of LXR to a peptide derived from a coactivator [66,67].

Prostaglandin F_2α_ (PGF_2α_) is one of the cyclooxygenase metabolites of arachidonic acid. PGF_2α_ antagonized the T0901317 induced activation of LXRα-LBD and LXRβ-LBD in a dose dependent manner with an IC_50_ value of 12.6 µM and 15 µM respectively. It also antagonized the activation of ABCA1 and ABCG1 promoter activity induced by T0901317 [68]. Small heterodimer partner interacting leucine zipper protein (SMILE) has been identified as a nuclear corepressor of the nuclear receptor (NRs) family. Ursodeoxycholic acid (UDCA), is a bile acid which increases the SMILE protein level in a dose dependent manner there by inhibits the LXRα [69]. 5α,6α-Epoxycholesterol (5,6-EC) is a product of cholesterol auto oxidation found in the human circulation and atherosclerotic lesions. In an LXR-cofactor interaction assay, 5,6-EC bound directly to LXR-LBD and disrupted the recruitment of a number of cofactors to both LXRα and LXRβ. 5,6-EC also exhibits the antagonist behavior with LXR-mediated genes [70].

### 2.3. Natural Products and Derivatives

#### 2.3.1. Natural Agonists

**Figure 4:**
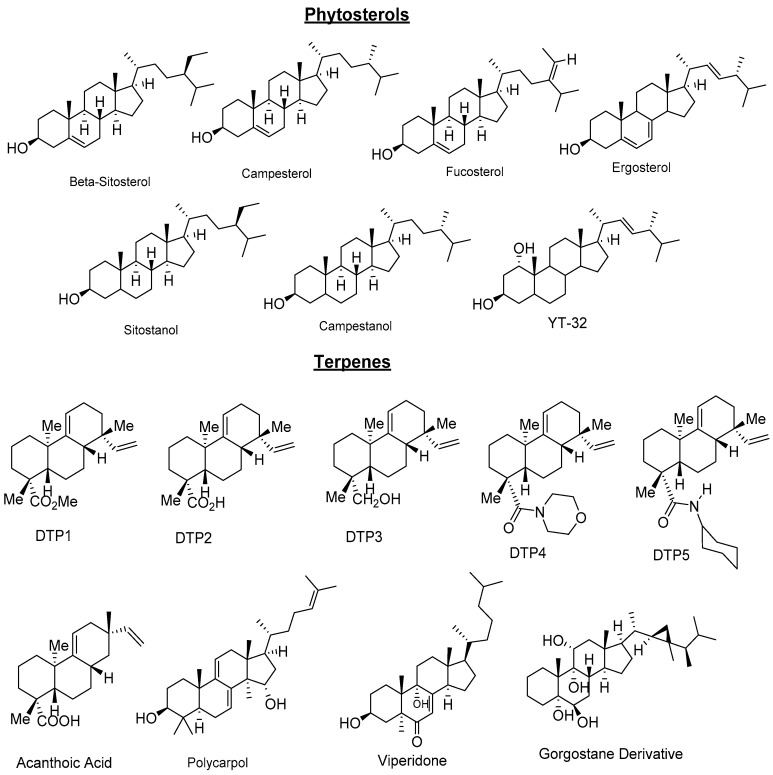
Selected phytosterols and terpenes as naturally occurring LXR ligands.

A variety of compounds purified from plants or fungi have been shown to modulate the activity of LXRs. These naturally occurring compounds could offer potential therapeutic efficacy while minimizing some side effects, such as hypertriglyceridemia [71]. Phytosterols including ergosterol, brassicasterol, campesterol, β-sitosterol, stigmasterol and fucosterol are naturally occurring sterols and are the plant equivalent of mammalian cholesterols (Figure 4). The treatment of intestinal cells with phytosterols increases the expression of LXR target genes [72], suggesting that phytosterols or their metabolites act as LXR ligands and influence cholesterol metabolism [73]. Stanols and sterols increases intestinal ABCA1 expression (sitostanol 244%, sitosterol 273%, campesterol 213%, fucosterol 166%) and decreases cholesterol absorption, suggesting that LXR is a target for dietary regulation of intestinal cholesterol metabolism [72,73]. However, a recent study has shown that dietary plant sterols and stanols inhibit cholesterol absorption within the intestinal lumen, which is independent of LXR [74]. The EC_50_ values of selected phytosterols are listed in Table 1 as compared to GW3965A, a widely used synthetic agonist of LXR.

Fucosterol, a sterol abundant in marine algae, has hypocholesterolemic effects and increases plasma high-density lipoprotein (HDL) activity. Fucosterol significantly induced the transactivation of both LXRα (+155% at 200 µM; *p* < 0.05) and LXRβ (+83% at 200 µM; *p* < 0.05) in HEK 293 cells [75]. In HepG2 cells, fucosterol (200 µM) increased ABCA1, ABCG1, ABCG5, ABCG8 and cholesteryl ester transfer protein (CETP) mRNA expression by 2.4-, 13.2-, 1.5-, 1.3- and 0.8-fold (*p* < 0.05) respectively.

YT-32 ((22*E*)-ergost-22-ene-1α,3β-diol), derived from ergosterol or brassicasterol, directly binds to LXRα and stimulates the interaction of LXRα with ACTR and DRIP205 at a 10 µM concentration. It also activates the LXRβ with an EC_50_ value of 1.1 µM. Unlike the synthetic LXR agonist T0901317, YT-32 inhibits intestinal cholesterol absorption without increasing plasma triglyceride levels. Thus, YT-32 selectively modulates intestinal cholesterol metabolism [76].

Diterpenes are natural steroids that are widely distributed in plants and insects (Figure 4 and Table 2). Acanthoic acid (AA) is a pimaradiene diterpene, isolated from the root bark of *Acanthopanax* *koreanum Nakai*. AA activates LXRα and LXRβ and modulates CCl_4_-induced liver fibrosis in animals by inhibiting NF-κB translocation. AA has also been found to inhibit growth of rat hepatic stellate cells (HSC-T6) via activation of LXR [77].

Traves et al. reported that stimulation of macrophages with acanthoic acid-related diterpenes (DTP 1-5) induces the expression of LXR target genes and cholesterol efflux to a similar level observed with synthetic agonists like GW3965 and T0901317 [78]. Using a scintillation proximity assay, acanthoic acid, polycarpol, gorgostane derivatives and viperidone derivatives selectively activate LXRα in HEK293 cells, as shown in Table 2 [79].

**Figure 5:**
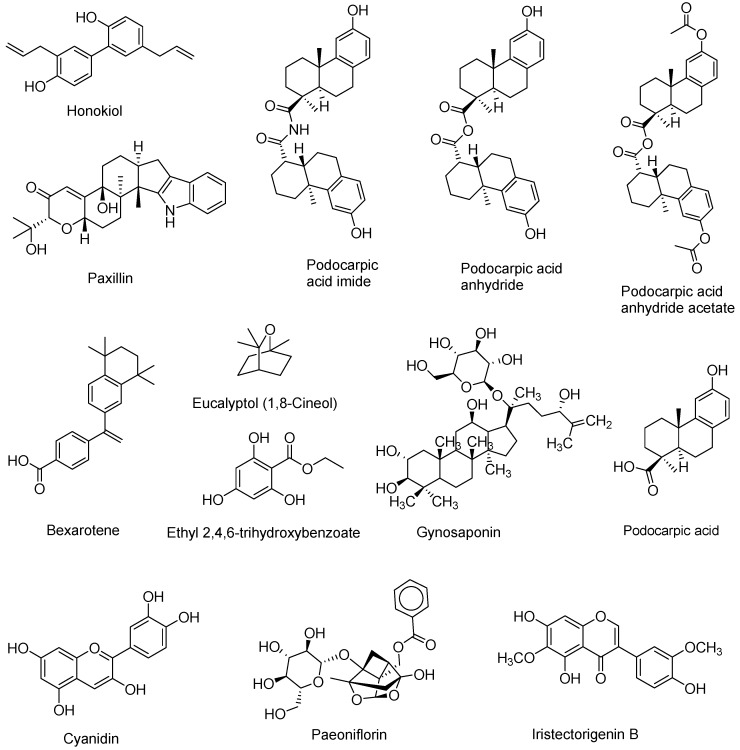
Structures of selected natural compounds as LXR ligands.

Several natural ligands isolated from herbal medicines (Figure 5) have also shown activities towards LXRs (Table 2). For example, gynosaponin TR1 ((20*S*)-2α,3β,12β,24(*S*)-pentahydroxydammar-25-ene 20-*O*-β-d-glucopyranoside), a dammarane saponin which is isolated from Chinese herbal medicine *Gynostemma pentaphyllum*, is an LXR agonist. It also exhibits selective activity towards LXRα over LXRβ. In HEK293 cells, gynosaponin TR1 induced a significant elevation of luciferase activity for LXRα at 10 µM concentration. It also enhanced the expression of ABCA1 and ApoE gene in THP-1 derived macrophages at the same concentration levels, which promotes the cholesterol efflux [80].

Podocarpic acid is a natural non-steroidal LXR agonist derived from plant resins [81]. From the LXR scintillation proximal binding assays it was concluded that podocarpic acid derivatives such as its dimer anhydride and imides binds to both LXRα and LXRβ at 1–2 nM concentrations. Cell based transactivation studies on HEK-293 cells indicate that the anhydride dimer exhibited the EC_50_ value of 1 nM against both receptors and showed 50- and 8-fold maximal induction of α and β LXR receptors, respectively. The more stable and potent imide increases the total plasma cholesterol levels by 28% with concomitant increase of HDL-cholesterol by 22% and decreases the LDL by 11% in hamsters. Similar results were also observed in mice where HDL-cholesterol levels were increased by 19% [82].

More recently, it has been shown that honokiol, extracted from the bark of Houpu (*Magnolia officinalis*), induces LXR transactivity in a reporter assay. It increases ABCA1 mRNA and protein levels in a dose-dependent manner in U251-MG cells and in THP-1 cells by 3 fold. Honokiol increases the ABCG1 and ApoE mRNA levels in THP-1 macrophage by 2.9- and 3-fold, and their protein levels by 4.5- and 7-fold, respectively [83]. Similarly, honokiol increases expression of the ABCA1 gene in peritoneal macrophages [84].

Paeoniflorin (*Paeonia lactiflora Pall*) is one of the active ingredients of Shaoyao, an herbal medicine with anti-hyperlipidemic, neuroprotective, and anti-hepatofibrosis effects. Reporter assays show that paeoniflorin transactivates the GAL4 promoter with an EC_50_ value of 8.7 µM. It also transactivates the PLTP promoter, ABCA1 promoter and rat CYP7A1 promoter with EC_50_ values of 21.6 µM, 11.9 µM, and 66 µM, respectively [85]. These results suggest that paeoniflorin may act as an LXRα agonist.

Iristectorigenin B, isolated from Shegan (*Belamcanda chinensis*), significantly induced the transcriptional activity of both LXRα (+540%) and LXRβ (+331%) at 20 µM in a dose-dependent manner. Iristectorigenin B increased cholesterol efflux to HDL and reduced cellular cholesterol concentration in macrophages. It also significantly increased the mRNA expression levels of both ABCA1 and ABCG1 LXR-responsive genes by 2.0 and 1.9-fold at 10 µM concentration, respectively [86].

Ethyl 2,4,6-trihydroxybenzoate (ETB) was isolated from *Celtis biondii* and was shown to directly bind to and stimulate the transcriptional activity of LXRα and LXRβ. ETB significantly induced the transactivation of both LXRα (+64%) and LXRβ (+55%) at 100 µM with an EC_50_ values of 80.76 and 37.8 µM, respectively. ETB increased the cholesterol efflux to HDL and reduced cellular cholesterol concentration in THP-1, RAW 264.7 macrophages and intestinal cells in a dose dependent manner. At a concentration of 100 µM, ETB increased ABCA1 mRNA expression by 7.4-fold for THP-1-derived macrophages and 2.1-fold for RAW 264.7 macrophages, respectively, without inducing lipid increase in HepG2 cells [87].

Cyanidin, a natural flavonoid found in many fruits and vegetables, is known to regulate cellular lipid metabolism. Cyanidin induced the transactivation of LXRα by 32% (at 50 µM), 59% (at 100 µM) and LXRβ by 33% (at 100 µM). The K_D_ values of cyanidin with LXRα and LXRβ were measured at 2.16 and 73.2 µM, respectively. Cyanidin activates the LXRα with an EC_50_ value of 3.48 µM and LXRβ at 125.2 µM. Cyaniding also activated LXR responsive genes including ABCA1, SREBP-1c and ABCG5 by 2.5 fold (100 µM), 3.6-fold (100 µM) and 1.4-fold (100 µM), respectively. It also reduced the concentrations of cellular TG by 21% and 23% in THP-1 and HepG2 cells, respectively at 100 µM concentration [88].

Cineole, a small aroma compound present in teas and herbs, has been shown to stimulate the transactivation of LXRs. Treatment of CHO-K1 cells with cineole induced the transactivation of LXRα by more than 75% and LXRβ by over 100%. In RAW 264.7 macrophages, cineole was able to reduce cellular cholesterol levels. Cineole also significantly increased the mRNA expression of the LXR responsive genes. Surprisingly, in hepatocytes that were stimulated with cineole, LXR responsive genes FAS, SREBP-1c and SCD-1 were markedly downregulated. These results suggest that cineole is acting like a partial agonist which selectively activates LXRs without inducing hepatic lipogenesis [89].

Apart from the plants and herbal derivatives, some fungal derivatives such as paxillin and ergostan-4,6,8,22-tetraen-3-one, an erostane derivative (isolated from Norwegian soil) have also been shown to possess agonist activities towards LXRs [90].

#### 2.3.2. Natural Antagonists

While the main focus of LXR ligand development in the past 10 years has been on therapeutically useful agonists, several naturally occurring antagonists have emerged in recent reports that demonstrated the ability to reduce triglycerides and improve fatty liver conditions, suggesting potential utility of LXR antagonists as therapeutic agents.

**Figure 6:**
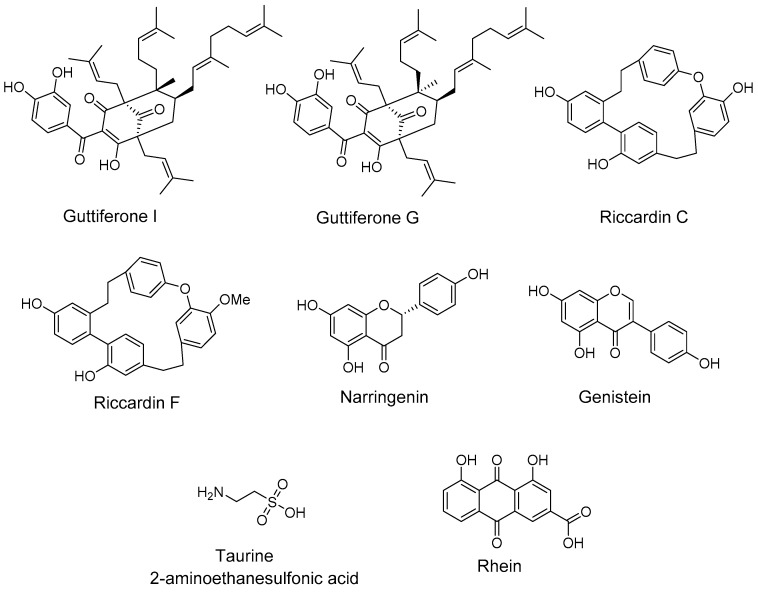
Structures of selected natural compounds as LXR antagonists.

Figure 6 shows a few naturally occurring compounds that act as LXR antagonists. Guttiferone inhibits the activity of LXRα with an IC_50_ value of 3.4 µM and that of LXRβ with an IC_50_ value of >15 µM, which indicates the 5-fold selectivity towards LXRα. However, guttiferone did not show any LXR activity in coactivation assays [91]. Riccardin C (RC) and riccardin F (RF) are non-sterol natural LXR antagonists isolated from liverwort *Blasia pusilla*. RC is a selective antagonist of LXRβ and can enhance ABCA1 expression and cellular cholesterol efflux in THP-1 macrophages by 2-fold, ABCG1 by 2.6-fold, SREBP-1c by 1.6-fold at 10 µM concentration. Riccardin F is a natural dual antagonist for both LXR isoforms [92].

Naringenin is a flavonoid that can be found in grapefruit, oranges and tomatoes. Goldwasser et al. confirmed that naringenin displayed antagonist activities towards LXRα in the presence of LXRα agonist T0901317. It inhibited the LXRα activity by 28.4% at 126 µM and 39.1% at 400 µM concentrations in HEK 293 T cells which were stimulated with 4.7 nM T0901317. It also reduced the abundance of mRNA of ABCA1, ABCG1, HMGR and FAS genes by 92%, 27%, 43%, and 41% respectively [93].

Genistein is a soy-derived flavone found to act as an LXR modulator. Like other isoflavones, genistein is believed to regulate LXR activity indirectly by promoting the phosphorylation. Genistein reduced the expression of SREBP-1c and ABCA1 by suppressing the activation of LXRα. At the same time it was shown to increase the expression of ABCG5 and ABCG8 by activating LXRβ, thus exhibiting opposing actions on the two different LXRs [94].

Taurine (2-aminoethanesulfonic acid), which is abundant in seafood, is known to exhibit the nutritional implications in hypercholesterolemia and atherosclerosis. Hoang et al. [95] reported that taurine binds directly to LXRα in CHO-K1 cells, and stimulates its transcriptional activity by +90% at 100 µM concentration. They also confirmed that, taurine reduced the cellular cholesterol and TG levels in hepatocytes by not inducing the fatty acid synthesis genes including FAS and SCD-1. Taurine achieved this via inhibiting the nuclear translocation of the sterol regulatory element-binding protein 1 protein (SREBP-1c) [95].

The Chinese herbal medicine Dahuang (*Rheum palmatum* L.) contains an anthraquinone-like ingredient, rhein (4,5-dihydroxyanthraquinone-2-carboxylic acid) that was discovered to exhibit antagonistic activities toward both LXRs [96]. The luciferase activity assays showed that rhein dose-dependently inhibited the transcriptional activity of LXRα and LXRβ stimulated by the agonist GW-3965. This suggests that rhein inhibits the expression of SREBP-1c or its target genes in the liver [96].

Kanaya et al. reported that the fatty acid biosynthesis pathways were downregulated in mouse livers treated with white button mushroom (WBM). The LXR luciferase activity was significantly decreased when cells were treated with T0901317 (LXR agonist) together with WBM extract. Furthermore, suppression of LXR signaling in HepG2 cells was also found to mediate the downregulation of FAS and ELOVL6 expression by WBM [97].

The selective inhibition of the transactivity of LXRβ in the presence of LXRβ agonist GW3965 by Kuding tea extract revealed its antagonist property. The mRNA expression of LXRβ targets genes including ABCA1, ABCG1, LPL and ApoE, were significantly inhibited in the liver and fat tissue in mice treated with the Kuding tea extracts [98]. Okra polysaccharide has also been known to reduce the LDL-c levels in obese C57BL/6 mice by suppressing the expression of LXR target genes such as ATP-binding cassette transporter G1, ApoE, and CYP7A1 [99].

Kim et al. reported that methanol extracts (MEH184 and MEH185) of *Parthenocissua tricuspidata* (*Virginia creeper*) and *Euscaphis japonica* (Korean sweetheart tree) inhibited the transcriptional activity of LXRα in the presence of both agonists T0901317 and 22(R)-HC. Both these extracts dramatically reduced the expression of FAS, ADD1/SREBP-1c and LXRα mRNA, which have been established to be LXRα target genes [100].

#### 2.3.3. Synthetic LXR Ligands

**Figure 7:**
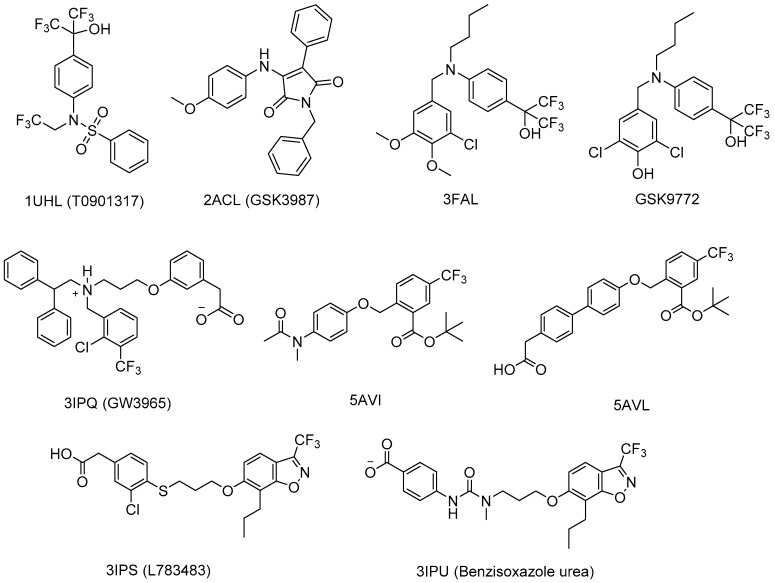
Structures of representative synthetic LXRα ligands.

Multiple findings proving LXR’s efficacy in various disease states have driven the development of novel and potent LXR modulators. T0901317 (Figure 7) is a non-steroidal synthetic ligand composed of a tertiary sulfonamide and a bistrifluoromethyl carbinol, allowing for vital hydrogen binding activity responsible for activating LXR and recruiting its cofactors [101]. The synthetic ligand has been proven to induce ABCA1 expression, decrease cancer cell proliferation, and downregulate amyloid-β (Aβ) peptide production. While T0901317 appears to be a very effective LXR activator, the ligand is also an activator of the pregnane X receptor (PXR) and the farnesoid x receptor (FXR), as well as an inhibitor of RORα and RORγ, putting the selectivity of this particular synthetic ligand into question [102,103]**.**

GW3965 is another non-steroidal, tertiary benzylamide shown to more selectively activate LXRα, with an EC_50_ value of 648 ± 179 nM for activating LXRα, according to Fradera et al. [104]. The ligand, developed by GlaxoSmithKline, has been tested for its benefits in the prevention of atherosclerotic incidences, ischemia-induced brain damage, and other arthritic and inflammatory incidences. While GW3965 provides these benefits with increased selectivity for the LXRα, the ligand has been associated with hypertriglyceridemic effects in mouse subjects, hindering possible therapeutic use [29,105].

T0901317 and GW3965 have been studied extensively in the activation of LXR, and as such, multiple research groups and companies have developed novel ligands to compare to the previous synthetic molecules in search for new potent chemical scaffolds to interact within the LXR ligand-binding pocket. GlaxoSmithKline has continued to develop these LXR activators with the synthesis of GSK3987. GSK3987 is a substituted 3-(phenylamino)-1*H*-pyrrole-2,5-dione, or a maleimide, that was proven to be a potent LXRα and LXRβ dual agonist and ABCA1 inducer, with an EC_50_ value of 40 nM in activating LXRβ. While the efficacy of the new ligand is nearly comparable to previously established LXR agonists, hypertriglyceridemia remains a concern for GSK3987 [106].

Chao et al. developed new N-phenyl tertiary amines based on modifications of T0901317. With one of the suggested ligands (3FAL) co-crystallized within LXRβ to expose possible regions of interaction within the receptor, GSK9772 was revealed to be a LXR modulator claimed to have anti-inflammatory activity, with a 10-fold selectivity for LXR-mediated transrepression of proinflammatory gene expression over transactivation of lipogenic signaling pathways without the accumulation of triglycerides that have plagued the previous synthetic ligands [107].

Fradera et al. compared the LXR binding of GW3965 to the binding of a benzisoxazole urea (crystallized in 3IPU) and F_3_methylAA/L783483, a benzisoxazole derivative (crystallized in 3IPS). This class of compounds was shown to be effective in treating inflammation and cardiovascular diseases, and the docking studies of these ligands presented the possible mechanism of cofactor signaling with LXR homodimers and LXR: RXR heterodimers [104]. A series of tert-butyl benzoate analogs, particularly with two (5AVI) and three (5AVL) aromatic rings has been synthesized for evaluation as LXR agonists. The ligand found crystallized in 5AVL was found to induce blood ABCA1 mRNA expression without affecting the plasma triglyceride levels in both mice and cynomolgus monkeys [108].

**Figure 8:**
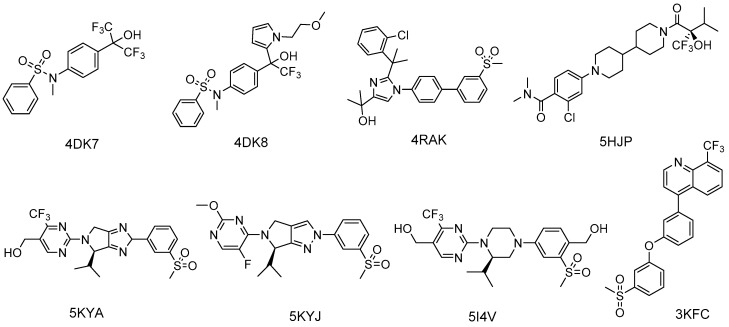
Structures of representative synthetic LXRβ ligands.

A new class of LXRβ partial agonists with the introduction of a pyrrole group to a T0901317 analog has been reported [109]. Two crystal structures, 4DK7 and 4DK8 (Figure 8), reveal that two separate orientations comparing these ligands appear to both seal off the ligand-binding site of LXRβ, confirming the receptor’s activation [9]. Bernotas et al. prepared 4-(3-aryloxyaryl)quinolines with sulfone substituents that proved to have high affinity for the LXR binding site, with a potency comparable to T0901317. Assays using J774 mouse cells revealed an increase in ABCA1 mRNA without PPAR activation, which was an unwanted effect in the Wyeth synthesized, quinoline-based LXR ligands [110]. Kick et al. developed selective LXRβ partial agonists containing either pyrazole or imidazole functional groups. The most potent ligand, crystallized in 4RAK, induces ABCA1 with an EC_50_ of 1.2 μM ex vivo in human blood, and no significant triglyceride elevation when tested in mice [111].

With the utilization of the structure-based drug design platform, Contour, Zheng et al. synthesized LXRβ agonists mainly containing a 2-(methylsulfonyl)benzyl alcohol and a piperazine core. One such compound (514V, Figure 8) exhibited 27 fold selectivity for LXRβ over LXRα, and is currently in clinical trials for the treatment of atopic dermatitis [112].

More recent studies have seen synthetic agonists that focus on the therapeutic potential associated with LXR activation and Alzheimer’s disease. Stachel et al. synthesized LXRβ selective agonists containing bispiperidine and an isopropyl trifluoromandelate group that, upon dosing in rats, allows for penetration into the brain, elevated apolipoprotein E and ABCA1 levels in the brain, and a noticeable decrease in Aβ peptide production, all without an effect on triglyceride levels [54]. LXR modulators synthesized by Tice et al. have also been tested to increase ABCA1 levels in the brain, with compounds 5KYA and 5KYJ crystallized in LXRβ. These particular compounds all contain a 2,4,5,6-tetrahydropyrrolo [3,4-c]pyrazole core, acting as a novel scaffold for these modulators [113].

## 3. LXR Ligand Design Considerations—A Molecular Modeling Perspective

The ligand-binding domains of the two LXR isoforms are very similar, with 74% sequence identity and have identical residues in the first layer of their binding pockets (i.e., residues that make direct contact with most ligands) [8]. In the following discussion we will focus on the structure of LXRβ, keeping in mind the strong structural similarity between the two forms of the protein. It should be noted that LXRα residue numbers are offset from those of LXRβ by negative 14 (e.g., Trp457 in LXRβ is Trp443 in LXRα).

**Figure 9:**
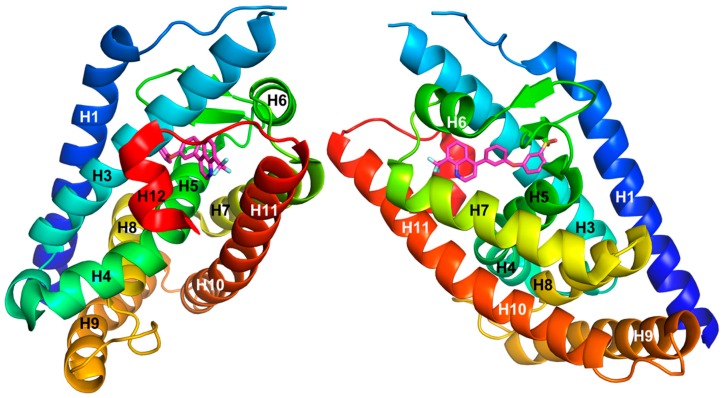
The 12 α-helices forming a three-layered sandwich fold in liver X receptors.

X-ray crystallography reveals the LXR structure to be consistent with other nuclear receptors, with 12 α-helices forming a three-layered sandwich fold (Figure 9) [101]. The volume of the binding pocket has been reported to be between 560–1090 Å^3^ [8,103], with this volume depending strongly on the size and character of the bound ligand. This wide range of reported volumes indicates the large degree of plasticity exhibited by LXR. This plasticity, along with the unusually large size of the LXR binding pocket—steroid receptor binding pockets generally have volumes in the range between 420–550 Å^3^—results in receptors that can accommodate ligands whose size and shape vary substantially. 

The mechanism of LXR activation is consistent with activation of other nuclear receptors, such as the much-studied estrogen receptor [8,102]. Upon agonist binding, helix 12, also known as activation function 2 (AF2), assumes a conformation that closes the binding pocket and creates a groove into which coactivator proteins can bind [114]. There is strong structural (crystallographic) evidence that closing of the binding pocket is mediated by an R-H···π interaction (R = C or N) that is established upon agonist binding between His435 (helix 11) and Trp457 (helix 12) [101].

**Figure 10:**
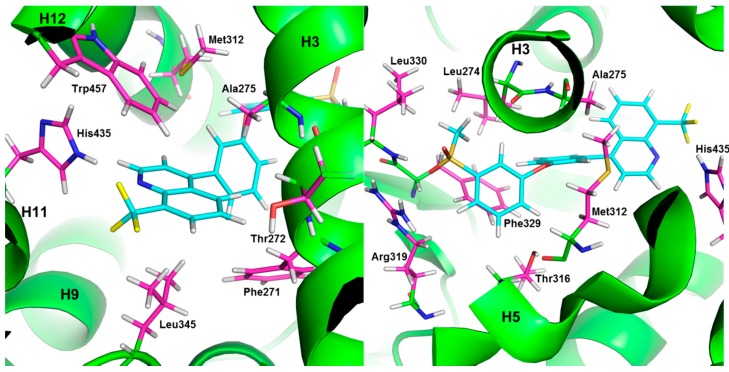
The binding pocket of LXRβ based on the optimized 3KFC crystal structure.

The binding pocket is largely hydrophobic with many non-polar aliphatic and aromatic residues located throughout, as illustrated in Figure 10. There is a hydrophilic region of the binding pocket possessing several polar and charged amino acids which is, because of its proximity to the protein surface, solvent accessible. This hydrophilic region is located near helices 1 and 5 and the β-sheet region found between helices 5 and 6. It should also be noted that within the hydrophobic region, on the side of the binding pocket directly opposite the hydrophilic region, are found the moderately polar His435 and Trp457 amino acids (helices 11 and 12) responsible for receptor activation.

Here we use density functional theory (DFT-D) binding energy calculations to determine the particular ligand-residue interactions that play large roles in stabilizing complexes of LXRβ with various agonist ligands. These calculations were carried out for seven LXRβ crystal structures obtained from the protein data bank (PDB), each crystal structure having a distinct agonist ligand. The seven PDB structures used in this study are 1PQC [103], 1PQ6 [103], 3KFC [110], 4DK7 [109], 4DK8 [109], 3L0E [115], and 5HJP [54] having resolutions between 2.3Å (3L0E) and 2.8Å (1PQC). Of particular note among these are the 1PQC and 1PQ6 structures, which contain synthetic ligands known to have strong agonist activity, T0901317 [116] and GW3965 [117] respectively. Amino acids within 4Å of the ligand in any one of the seven crystal structures were considered for the DFT-D study, with a total of 40 amino acids fitting this criterion.

All pairwise binding energy calculations were performed at the BLYP-D/def2-TZVP [118] level of theory using the ORCA molecular electronic structure package [119] through the Cuby4 interface [120]. The COSMO implicit solvation method, along with a dielectric constant of 4.0, was used to mimic the protein’s interior environment [121]. This approach has been adopted in several previously reported studies [122,123]. Here the simplest capping scheme of adding hydrogen atoms to amino acid backbone nitrogens and β-carbons was employed. In order to minimize the effects of steric clashes, the side chains of all structures were optimized using the simulated annealing method with backbone atom positions held fixed. These optimizations were carried out with the Yasara molecular modeling suite using the Amber99 force field (http://www.yasara.org).

**Figure 11:**
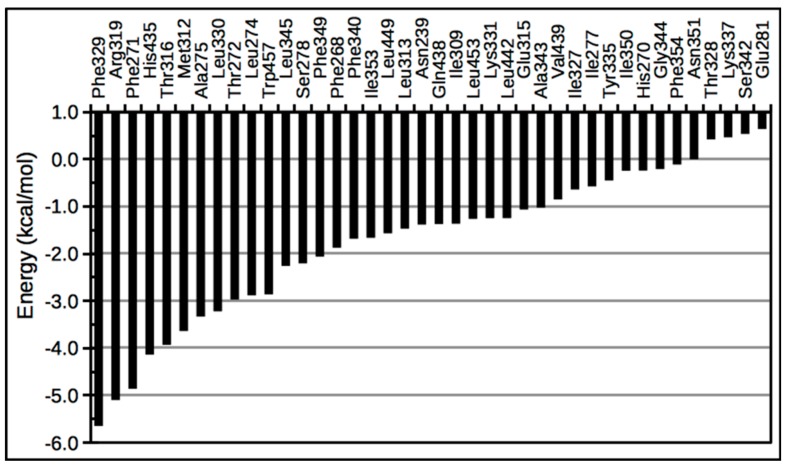
Pairwise interaction energies of 40 amino acids with ligand within the LXRβ binding pocket.

Figure 11 gives pairwise interaction energies of all 40 ligand-residue complexes averaged across all seven crystal structures. Here it is seen that there are 11 particularly strong interactions, those involving Phe329—Trp457 (in the order shown in Figure 11), exhibiting average interaction energies of about 3.0 kcal/mol or stronger. Among these 11 interactions, those involving Phe329 (−5.66 kcal/mol), Arg319 (−5.11 kcal/mol), Phe271 (−4.87 kcal/mol), and His435 (−4.15 kcal/mol) are particularly strong. Interestingly two of these interactions, those associated with Phe329 and Phe271 are completely hydrophobic interactions while two are polar interactions, namely interactions involving the positively charged Arg319 and the heterocyclic ring of His435. It should be noted that Arg319 and His435 are the only two polar residues among the 11 discussed here. Arg319 is one of the residues located in the hydrophilic region of the binding pocket and is found near the protein surface. As noted above, the establishment of a strong contact between His435 and Trp457 is believed to play a role in ligand-mediated LXR agonism. It seems reasonable to hypothesize that direct interactions of the ligand with these two amino acids play a strong role in stabilizing the active conformation of the receptor, although it should be kept in mind that other ligand-residue or ligand-residue-residue interactions may play roles in changing the protein’s conformation.

There are 16 ligand-residue interactions, Leu345—Ala343, with moderate interaction energies, in the range from 1.0 kcal/mol to 2.5 kcal/mol. Most of these residues are aromatic or aliphatic amino acids that form dispersion-type interactions with the ligand, with the exceptions being Ser278 (neutral polar, −2.21 kcal/mol), Gln438 (neutral polar, −1.38 kcal/mol), Lys331 (positive, −1.26 kcal/mol), and Glu315 (negative, −1.07 kcal/mol). Nine residues, Val439—Asn351, form only weak interactions with the ligands, with interaction energies less than 1.0 kcal/mol. Four residues, Thr328, Lys337, Ser342, and Glu281, form mildly repulsive interactions with the ligands. The strongest repulsion occurs for Glu281, with an interaction energy of +0.63 kcal/mol. It should be kept in mind that all calculations are based on crystal structures in which only side chain (but not backbone) atoms are optimized, thus it is not very surprising to encounter these mildly repulsive interactions, which are most likely caused by steric clashes related to backbone atoms. 

**Figure 12:**
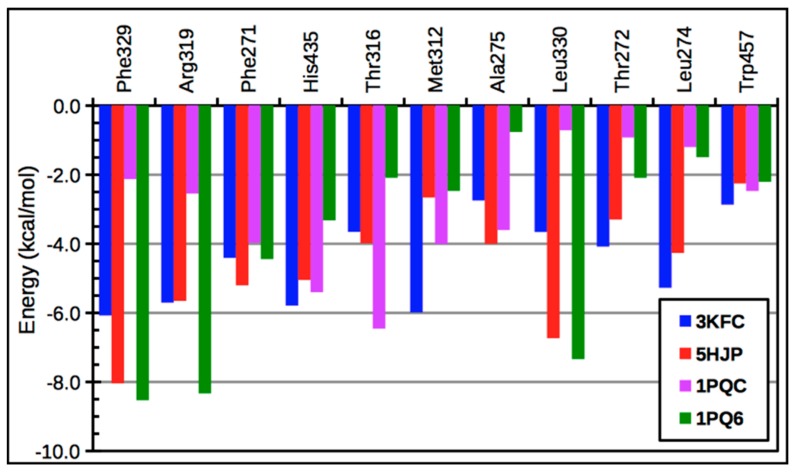
Interaction energies of 11 selected amino acids with ligands in the binding pocket of LXRβ.

Shown in Figure 12 are interaction energies for the 11 strongest ligand-residue interactions, with four different ligands, those found in 3KFC, 5HJP, 1PQC (T0901317), and 1PQ6 (GW3965). The most notable aspect of the data depicted here is that the only ligand-residue interactions that appear to be particularly strong for all four systems are the ones involving Phe271 (~4–5 kcal/mol) and His435 (~3.5–6 kcal/mol). Interactions for the 1PQC structure are notably weak for several of the residues here, which is perhaps not surprising when it is taken into consideration that, although it is known to be a strong agonist, T0901317 is the smallest of the ligands considered in this study. When the 1PQC structure is neglected, it is seen that all ligand-residue interactions, with the only other exceptions being 1PQ6/Ala275 and 1PQ6/Leu274, have a binding energy of at least two kcal/mol, meaning that each of these interactions make significant contributions to ligand binding.

As shown in Figure 10, the optimized 3KFC crystal structure is taken as an example of a typical interaction between LXRβ and a ligand, as this structure most consistently exhibits strong ligand-residue interactions for the 11 amino acids considered in Figure 12. Here the hydrophobic nature of the binding pocket can be seen, with Phe329, Phe271, Met312, and Leu330 being major points of contact and largely dictating the (hydrophobic) nature and shape of the central region of the binding cavity, as reflected by the (generally) strong interactions exhibited by these residues. One of the most surprising results shown in Figure 12 is the strong attraction between ligands and Ala275, also located in the central region of the binding pocket. Given its small size, it would generally be supposed that this amino acid would not form strong van der Waals contacts with ligands. Here it is seen that Ala275 establishes a strong dispersion contact with the ligand bicyclic group. In the solvent-accessible region of the binding pocket, containing Arg319 and Thr316, a strong interaction is formed between the positive Arg319 side chain and the ligand’s electronegative sulfonyl group. It should be noted that a sulfonyl group is commonly found in LXR ligands and generally is oriented towards this hydrophilic region of the binding pocket.

The important interactions involving the ligand, His435, and Trp 457 are also seen in Figure 10. Here a C-H…π interaction is established between the histidine imidizole ring and the heterocyclic ring of the tryptophan indole structure. It is believed that the establishment of this interaction, establishing contact between helix 12 (AF2) and helix 11, is responsible for LXR agonism. In terms of interactions of the ligand with these residues, there is clearly a N-H···F hydrogen bond between His435 and the ligand CF_3_ group and a π-π contact between the ligand and Trp457. It should be noted that there is also a strong possibility for a (stronger) N-H···N type hydrogen bond to be established between His435 and the ligand, and that this type of interaction might also exist. 

**Figure 13:**
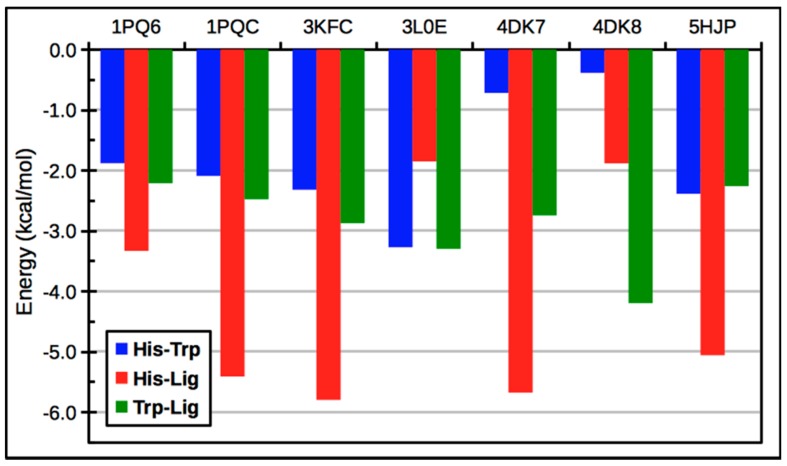
Calculated interaction energies for His435-Trp457, ligand-His435, and ligand-Trp457 interactions.

Figure 13 shows interactions energies for His435-Trp457, ligand-His435, and ligand-Trp457 contacts. The most interesting aspect of the data depicted here is the fact that the His435-Trp457 interaction energies are fairly strong (~2.0 kcal/mol or higher) for all structures except 4DK7 and 4DK8, which both represent partial agonists (the other five structures contain full agonists), which both have binding energies that are significantly lower. This finding supports the hypothesis that His435-Trp457 interactions mediate LXR agonism. The relationship between ligand-residue interactions and LXR agonism is not so clear. For example, ligand-His435 interactions are strong (above 3.0 kcal/mol) for five of the structures, but are significantly weaker for 3L0E (full agonist) and 4DK8 (partial agonist). Ligand-Trp457 interactions are all in the range from 2.0–4.0 kcal/mol, with no particular preference for full agonists above partial agonists.

## 4. Summary

Concurrent with our increasing knowledge of the roles of LXRs in lipid homeostasis, development of selective and potent ligands of the hormone receptors has gained significant ground toward clinical applications of LXR modulations. As LXRs are now known as key regulators of the reverse cholesterol transport pathway, activation of LXRs via agonist ligands represents a viable approach to controlling cholesterol flux and thereby preventing and treating cardiovascular diseases. LXRs also play central roles in lipogenesis and maintenance of glucose balance in systemic circulation, presenting potential opportunities for treatment of type 2 diabetes and metabolic disorders. However, LXR activation also promotes triglyceride synthesis in the liver and elevates triglyceride levels in plasma, presenting a major challenge in the development of LXR agonists for cardiovascular disease intervention. On the other hand, the dominant expression of LXRα in the liver provides a rationale to develop LXRβ-selective ligands so as to minimize the lipogenic effects. In this light we examined the interaction energies of ligands and the important amino acid residues in the LXRβ ligand binding domain. We believe that such multitude of interactions should be taken into consideration in designing more potent molecules that bind to the LXRβ receptor with greater selectivity and specificity. 

## Figures and Tables

**Table 1:** EC_50_ values of selected phytosterols evaluated in a coactivator peptide recruitment assay.

Phytosterol	EC_50_ (nM)	
	LXRα	LXRβ
Sitosterol	42	26
Campesterol	43	28
Fucosterol	33	42
Sitostanol	136	110
Campestenol	122	124
GW3965A	197	41

**Table 2:** LXR activities of diterpenoids, steroids and triterpenoids.

Name	LXR SPA Binding IC_50_ (µM)		Cofactor Association HTRF * Assay, EC_50_ (µM)		Transactivation Max. Fold Induction	
	LXRα	LXRβ	LXRα	LXRβ	LXRα	LXRβ
Acanthoic acid	0.25	1.49	0.18	≥50	15.9 (100 µM)	5.6 (100 µM)
Viperidone	0.10	---	≥15	-----	----	----
Polycarpol	0.12	≥15	0.030	≥50	-----	-----
Gorgostone Derivative	0.07	0.2	0.05	----	13 (10 µM)	2.2 (10 µM)

* HTRF assay: Homogeneous Time Resolved Fluorescence assay.
